# A Comparison of the Gene Expression Profiles of Non-Alcoholic Fatty Liver Disease between Animal Models of a High-Fat Diet and Methionine-Choline-Deficient Diet

**DOI:** 10.3390/molecules27030858

**Published:** 2022-01-27

**Authors:** Mohammed Abdullah Alshawsh, Abdulsamad Alsalahi, Salah Abdalrazak Alshehade, Sultan Ayesh Mohammed Saghir, Ahmad Faheem Ahmeda, Raghdaa Hamdan Al Zarzour, Ayman Moawad Mahmoud

**Affiliations:** 1 Department of Pharmacology, Faculty of Medicine, University of Malaya, Kuala Lumpur 50603, Malaysia; ahmedsamad28@yahoo.com; 2 Discipline of Pharmacology, School of Pharmaceutical Sciences, Universiti Sains Malaysia (USM), Gelugor 11800, Malaysia; salah_alsh@outlook.com (S.A.A.); raghdaa@usm.my (R.H.A.Z.); 3 Department of Medical Analysis, Princess Aisha Bint Al-Hussein College of Nursing and Medical Sciences, Al-Hussein Bin Talal University, Ma’an 71111, Jordan; sultan.s.ayesh@ahu.edu.jo; 4 Department of Basic Medical Sciences, College of Medicine, Ajman University, Ajman P.O. Box 346, United Arab Emirates; a.ahmeda@ajman.ac.ae; 5 Center of Medical and Bio-Allied Health Sciences Research, Ajman University, Ajman P.O. Box 346, United Arab Emirates; 6 Physiology Division, Zoology Department, Faculty of Science, Beni-Suef University, Beni-Suef 62514, Egypt; ayman.mahmoud@science.bsu.edu.eg

**Keywords:** animal models, gene expression patterns, non-alcoholic fatty liver disease, non-alcoholic steatohepatitis, high-fat diet, methionine-choline-deficient diet, liver fibrosis, signaling pathway

## Abstract

Non-alcoholic fatty liver disease (NAFLD) embraces several forms of liver disorders involving fat disposition in hepatocytes ranging from simple steatosis to the severe stage, namely, non-alcoholic steatohepatitis (NASH). Recently, several experimental in vivo animal models for NAFLD/NASH have been established. However, no reproducible experimental animal model displays the full spectrum of pathophysiological, histological, molecular, and clinical features associated with human NAFLD/NASH progression. Although methionine-choline-deficient (MCD) diet and high-fat diet (HFD) models can mimic histological and metabolic abnormalities of human disease, respectively, the molecular signaling pathways are extremely important for understanding the pathogenesis of the disease. This review aimed to assess the differences in gene expression patterns and NAFLD/NASH progression pathways among the most common dietary animal models, i.e., HFD- and MCD diet-fed animals. Studies showed that the HFD and MCD diet could induce either up- or downregulation of the expression of genes and proteins that are involved in lipid metabolism, inflammation, oxidative stress, and fibrogenesis pathways. Interestingly, the MCD diet model could spontaneously develop liver fibrosis within two to four weeks and has significant effects on the expression of genes that encode proteins and enzymes involved in the liver fibrogenesis pathway. However, such effects in the HFD model were found to occur after 24 weeks with insulin resistance but appear to cause less severe fibrosis. In conclusion, assessing the abnormal gene expression patterns caused by different diet types provides valuable information regarding the molecular mechanisms of NAFLD/NASH and predicts the clinical progression of the disease. However, expression profiling studies concerning genetic variants involved in the development and progression of NAFLD/NASH should be conducted.

## 1. Introduction

As a term, non-alcoholic fatty liver disease (NAFLD) was proposed to describe the histopathological fatty changes in hepatocytes in which alcohol consumption is not involved as the etiological inducer [1]. In general, NAFLD embraces several forms of liver disorders involving fat disposition in hepatocytes ranging from the simple steatosis to the advanced stage, namely, non-alcoholic steatohepatitis (NASH) [2]. Moreover, NAFLD is known as the hepatic manifestation of metabolic syndrome since it is associated with metabolic disorders, such as obesity, type 2 diabetes mellitus, dyslipidemia, and insulin resistance [1]. NASH is characterized by the finding of hepatic steatosis with inflammation due to the excessive disposition of fats in hepatocytes, which makes hepatocytes predisposed to oxidative stress and the subsequent inflammatory and fibrosis cascades [3]. However, obesity or simple hepatic steatosis is normally not sufficient to develop inflammation and fibrosis, thus the “second-hit” hypothesis was proposed to further overstate the liver injury [4]. On the other hand, NAFLD is considered a multisystem disease as it is significantly related to extra-hepatic complications, including type 2 diabetes, chronic kidney disease [5], cardiovascular disease [6], and even neurological diseases [7]. These complications make it difficult to develop a comprehensive experimental model that fully mimics the underlying mechanisms of NAFLD. Depending on the study objectives, many models have been developed to elucidate the pathophysiological mechanisms of NAFLD, including genetically modified animals, such as leptin deficiency (ob/ob) mice [6], leptin receptor (LepR) deficiency (db/db) mice [8], KK-A^y^ mice [9], phosphatase and tensin homolog (PTEN)-deficient mice [10], CD36-deficient mice [11], peroxisome proliferator-activated receptor alpha (PPAR-α) knockout mice [12], acyl CoA oxidase (AOX)-null mice [13], methionine adenosyltransferase-1A (MAT1A)-null mice [14], nuclear factor erythroid 2-related factor 2 (Nrf2)-deficient mice [15], Zucker (fa/fa) rats [16], Otsuka Long-Evans Tokushima Fatty (OLETF) rats [17], and Koletsky f/f rats [18]. Furthermore, NAFLD can be induced by feeding mice different kinds of diets, including a high-fat diet (HFD) [19], high-carbohydrate diet (HCD) [20], methionine- and choline-deficient (MCD) diet [21], and fast food (FF) diet [22]. Furthermore, to reach the NASH and fibrosis stage, some models need to be triggered by a second stimulus or agent (“second hit”), such as tunicamycin [23], dexamethasone [24], and carbon tetrachloride (CCl_4_) [25]. It was found that male C57BL/6J mice fed a high-fat, -sucrose, and -cholesterol diet with high fructose or glucose water and injected with CCl_4_ showed the closest similarity to the human NAFLD pattern [26]. However, the most common dietary animal models used to elucidate the molecular and cellular progression of NAFLD either induce hepatotoxicity with MCD or over-nutrition disorders with HFD [4] as both cover most NAFLD manifestations. Although a significant number of studies regarding the cellular and molecular pathogenicity of NAFLD/NASH have been conducted on those two dietary animal models, gene expression could provide an extra essential approach to elucidate the complex pathogenesis of NAFLD. Consequently, the current review aims to outline the differences in the gene expression profiles between HFD- and MCD diet-fed animal models, which can provide valuable information as both models represent a different manifestation to obtain a comprehensive understanding of the progression of NAFLD/NASH.

## 2. Challenges Encountered in NAFLD Animal Models

In humans, NAFLD is the outcome of a complex of genetic and environmental factors [9]. Since NAFLD is a polygenic disease, a full understanding of its pathogenicity has still been limited because of the genetic heterogeneity within populations, the suppression of a certain gene by another [27], and ethical issues regarding exposing humans to the whole experimental approaches and techniques during the study of NAFLD compared to what animals are exposed to [28]. For such reasons, several animal models were developed to achieve a better understanding of NAFLD. Firstly, relative similarities regarding physiological, metabolic, and anatomical features exist between humans and animals. Secondly, animal models provide a valuable opportunity to study the pathogenesis of NAFLD in a single homogeneous population since the effects of variations in age, genetic heterogeneity, gender, and diet are minimized in animal models [27]. In addition, the safer and easier collection of liver biopsies is an advantage of animal models over humans [28]. Nonetheless, an animal model that successfully reflects human NASH should feature the pathological profile of NASH, such as steatosis, inflammation, and fibrosis [3]. The latter fact directs us to another challenge: how to distinguish between bland steatosis and steatohepatitis. Simple steatosis and steatohepatitis share similar metabolic determinants, which supports the idea of looking for clear-cut metabolic boundaries to distinguish steatosis from steatohepatitis [28] and which animal model could solve such a challenge or achieve the transition from simple steatosis to steatohepatitis exactly as it develops in humans [28]. It has been reported that bland steatosis is well recognized by the presence of fatty infiltration while steatohepatitis involves the presence of both fatty infiltration and inflammation [29].

Regardless of the pathogenesis of NASH, over-nutrition is the critical factor responsible for the development of NAFLD since it leads to the most common metabolic disorders, namely, insulin resistance, glucose intolerance, obesity, and dyslipidemia, which are considered risk factors for the establishment of NASH [28]. Simple steatosis usually develops after insulin resistance, which results in a reversible cumulative disposition of fat in hepatocytes due to increases in lipid delivery to hepatocytes and the uptake of lipids by hepatocytes, an increase in the biosynthesis of triglycerides and fatty acids in the cytoplasm of hepatocytes, failure in the biosynthesis of very-low-density lipoprotein, and the export of triglycerides and the impairment in β-oxidation of hepatic mitochondria. To achieve the transition of steatosis to NASH, the combination of oxidative stress, lipid peroxidation, cell death, and proinflammatory cytokine-mediated liver injury constitutes the second hit [27]. Therefore, many etiological pathways are involved in the development of NASH, including mainly oxidative stress, lipid peroxidation, and inflammation [30]. Although the genetically altered ob/ob mice is the most common model used for studying obesity and NAFLD, it does not acquire NASH spontaneously and requires a second hit with lipopolysaccharide to trigger inflammatory events [31].

HFD- and MCD-fed animal models were found to be appropriate models for the study of NAFLD/NASH for humans since the HFD-fed animal model can mimic the metabolic abnormalities of NAFLD and other spectra of oxidative stress and inflammation but is unable to reach advanced stages, such as fibrosis and cirrhosis [4]. The MCD-fed animal model provides the histological hallmark of NASH because of its vulnerability to transition from simple steatosis to steatohepatitis and can reach fibrosis stages [4]. Furthermore, the HFD model develops insulin resistance [32] unlike the MCD-fed model, which shows lower insulin resistance levels [33]. Further, the HFD-fed model cannot mimic the whole features of histological changes that are associated with NASH as in humans while the MCD model fails to establish the transition from NAFLD (simple steatosis) to the advanced stage (NASH) [34]. Interestingly, the histological changes in the liver can be recovered among HFD-fed animals but not among MCD-fed animals after stopping the tested diet [35]. Table 1 shows a comparison between the diet models. However, the effects may differ depending on the mice strains, for example, feeding MCD to C57BL/6 mice [36], FVB/NJ mice [33], and db/db mice [37] resulted in noticeably lower serum insulin levels compared to chow diet-fed mice, but db/m mice showed a slight increase in serum insulin levels [37]. Moreover, to emphasize the important difference between intracellular oxidative stress caused by different types of diet, a recent study compared the mitochondrial (mt)DNA content between two mice models. A significantly lower mtDNA copy number was found in the MCD group compared to the HFD group, which could be attributed to the upregulation of both mitochondrial biogenesis and degradation-related genes. Interestingly, after stopping feeding, the MCD-fed group showed irreversible and consistently low levels of mtDNA, unlike the HFD group, which recovered to similar levels to those observed in the control chow-fed mice [38]. This finding justifies the increase in body weight with time and fat accumulation without fibrosis among the HFD group while a decrease in body weight and steatohepatitis with fibrosis was observed among the MCD group [35]. In general, high liver steatosis results in a higher average mtDNA copy number [39] while high liver steatohepatitis results in lower mtDNA copy numbers [40].

## 3. Molecular Signaling Pathways of NAFLD/NASH

The changes in the gene expression profile of NAFLD associated with HFD or MCD-fed models among different studies are discussed and categorized into four different molecular pathways, namely, lipid metabolism, inflammation, oxidative stress, and fibrogenesis. Moreover, the expressions of the key genes encoding for these four pathways are discussed in detail in the following sections.

### 3.1. Lipid Metabolism Pathway

Studies showed that HFD could induce either up- or downregulation of the expression of genes encoding proteins or enzymes that are involved in lipogenesis [42,43,44,45,46,47]. In addition, upregulation was associated with genes encoding hepatic cholesterol [44], leptin [46], and fatty acid biosynthesis [42,44,45] while downregulation was associated with genes encoding proteins or enzymes involved in hepatic β-oxidation [42,44,45], cholesterol clearance [42,43], and insulin resistance [30,47]. Moreover, the onset of changes in gene expression was delayed, i.e., 8–24 weeks after exposure to HFD (Table 2). Such changes in the gene expression of the lipid metabolism pathway indicated that feeding animals a HFD for a longer period could primarily induce simple steatosis with insulin resistance similar to the early stage of NAFLD in humans. On the other hand, feeding animals an MCD diet could induce lipogenesis to a limited extent as evidenced by the overexpression of a limited number of genes encoding proteins or enzymes involved in lipogenesis [48,49]. In addition, it seems that changes in the gene expression of lipid metabolism pathways were more restricted to adipocytes rather than hepatocytes. Moreover, the onset of changes in such genes involved in the lipid metabolism pathway occurred much earlier than in the HFD animal model (Table 2).

### 3.2. Inflammatory Pathway

It seems that most key regulatory genes involved in the inflammation pathway are upregulated by HFD following the initiation of lipogenesis [22,44,45,50]. Particularly, TNF-α was found to be the gene that was expressed earlier compared to the other genes encoding other inflammatory markers (Table 3). Similarly, genes encoding proteins involved in the inflammation pathway are upregulated by the MCD diet (Table 3). However, the gene expression of inflammatory markers in the MCD diet animal model occurs much earlier than that in the HFD animal model.

### 3.3. Oxidative Stress Pathway

Genes encoding proteins or enzymes involved in oxidative stress pathways are subjected to either up- or downregulation by HFD. Generally, genes encoding antioxidant enzymes, such as GPX1 and CAT, are downregulated by HFD [30] while genes encoding proteins and enzymes involved in the oxidative stress pathway are upregulated [22,44,45] (Table 4). In addition, such changes in the gene expression of oxidative stress occur mostly after a longer period of exposure to HFD (16–25 weeks). On the other hand, few studies have shown that the changes in the expression of genes encoding proteins involved in oxidative stress are more prominent in the MCD diet animal model (Table 4).

### 3.4. Fibrogenesis Pathway

NASH with fibrosis is characterized by extensive accumulation of the extracellular matrix (ECM), which is usually followed by extensive tissue damage [57]. The fibrogenesis process in the liver is thought to mainly be regulated by hepatic stellate cells [58], and its activation produces collagen I (COL I), collagen III (COL III), and transforming growth factor-β (TGF-β) [4] in response to overexpression of X-box-binding protein 1 (XBP1) [59], NACHT, leucine-rich-repeat, pyrin domain-containing protein 3 (NLRP3) [60], and platelet-derived growth factor (PDGF) [57]. Collagen is the principal component of the extracellular matrix or scars. A recent study showed that NASH with fibrosis is associated with upregulation of hepatic fibrillar collagen genes, namely, COL I and COL III [61]. Most studies on animals fed an HFD diet for a duration of less than 4 months showed that there were no significant changes in the expression of genes encoding proteins involved in the fibrogenesis pathway; however, such changes were found to be significant in studies with a longer duration from 24-25 weeks of exposure to HFD [22,45] (Table 5). On the other hand, the MCD diet was able to induce significant changes in the expression of genes that encode proteins involved in the fibrogenesis pathway much earlier than HFD and most of the related genes, such as COL1A1, COL1A2, MMP-9, MMP-13, TIMP-1, and TGF-β, were upregulated within 2 weeks of feeding with the MCD diet [51,52] (Table 5).

## 4. Conclusions

In conclusion, both models reflect different changes in the expression of genes encoding proteins and enzymes involved in several pathways of NAFLD/NASH pathogenesis. The MCD diet animal model can spontaneously develop liver injury characterized by a fibrosis pattern in a short period while the HFD animal model requires a longer duration of feeding with HFD to stimulate the progression of steatosis to mild steatohepatitis. In this study, it is clear that the difference between the two dietary animal models may be related to the differences in the animal strains and as a result, it might also be associated with differences in the genetic makeup of individual patients. The variation between HFD- and MCD diet-fed animal models in the patterns of gene expression involved in the development of NAFLD and steatohepatitis provides an insight into the molecular mechanisms of NAFLD/NASH and could help to find a selective target for an effective treatment. Further expression profiling studies concerning the genetic variants involved in both the development and progression of NAFLD/NASH using suitable animal models should be conducted.

## Figures and Tables

**Table 1:** A comparison between the NAFLD model mice fed MCD and HFD.

Comparison		HFD *	MCD **	HFD & MCD ***
Body weight		Higher	Lower	No change
Liver-to-body weight ratio		No change	Slightly lower	Higher
Serum biomarkers	TC	Higher	Lower	Lower
	TG	Slightly lower	Lower	Lower
	AST	Lower	Higher	NA
	Glucose	Higher	Lower	Slightly higher
	Insulin	Higher	Lower	Lower
Steatosis		Higher	Higher (but not as high as HFD)	Higher
Fibrosis		No change	Higher	No change
Inflammation lobular		Slightly higher	Higher	Higher
Hepatocellular ballooning		Slightly higher	Slightly higher	No change

* Compared (HFD 60% of energy as fat) to a standard chow diet as control ([22]); ** Compared to a standard chow diet as the control ([33,35,36]); *** Compared to an L-amino acid rodent diet as the control ([41]). TC: total cholesterols; TG: total triglycerides; AST: alanine aminotransferase; NA: not available.

**Table 2:** Gene expression profiles of hepatic lipid metabolism in HFD and MCD diet-fed animal models.

Gene Symbol	Nomenclature	Role	Diet Type	Duration (Weeks)	Animal Model	Gene Expression	Reference
ADIPOQ	Adiponectin, C1Q, and collagen domain containing	Required for normal glucose and fat homeostasis	HFD	8	Female SD rats	Down	[30]
ADIPOR1	Adiponectin receptor 1	Required for normal glucose and fat homeostasis	MCD	3	C57BL/6J mic	Up	[48]
ADIPOR2	Adiponectin receptor 2	Required for normal glucose and fat homeostasis		5	Male C57BL/6N mice	Up	[49]
AMPKα2	5′AMP-activated protein kinase catalytic subunit alpha-2	Inhibits protein, carbohydrate, and lipid biosynthesis		5	Male C57BL/6N mice	Up	
CPT-1A	Carnitine palmitoyltransferase-1 alpha	Mitochondrial oxidation of long-chain fatty acids	HFD	24	Male C57BL/6J mice	Down	[45]
				16	Male Wistar rats	Down	[44]
CYP4A10	Cytochrome P450, family 4, subfamily A, polypeptide 10	Involved in the metabolism of fatty acids	MCD	3	C57BL/6J mice	Down	[48]
CYPA14	Cytochrome P450, family 14, subfamily A	Involved in the metabolism of fatty acids		3	C57BL/6J mic	Up	
L-FABP	Liver-type fatty acid-binding protein	Plays a role in lipoprotein-mediated cholesterol uptake		4	db/db mice	Down	[37]
				4	db/m mice	Down	
FATP-1	Long-chain fatty acid transport protein 1	Mediates the ATP-dependent import of long-chain fatty acids into the cell		4	db/m mice	Up	
					db/db mice	Up	
				3	C57BL/6J mice	Down	[48]
				4	db/m mice	Up	[37]
FATP-2	Very long-chain acyl-CoA synthase	Activates long-chain and very-long-chain fatty acids					
					db/db mice	Down	
FATP-3	Solute carrier family 27 member 3	Acyl-CoA ligase activity for long-chain and very-long-chain fatty acids			db/m mice	Up	
					db/db mice	Down	
FATP-4	Long-chain fatty acid transport protein 4	Involved in the translocation of long-chain fatty acids across the plasma membrane			db/m mice	Up	
					db/db mice	Up	
				3	C57BL/6J mice	Up	[48]
FATP-5	Bile acyl-CoA synthase	Catalyzes the activation of bile acids via the formation of bile acid CoA thioesters		4	db/m mice	No change	[37]
					db/db mice	Down	
FASN	Fatty acid synthase	Catalyzes the de novo biosynthesis of long-chain saturated fatty acids	MCD	4	db/db mice	Down	
					db/m mice	Down	
			HFD	9	Male SD rats	Up	[42]
				24	Male C57BL/6J mice	Up	[45]
G3PDH	Glycerol-3-phosphate dehydrogenase	Glycolysis	HFD	8	Male Wistar rats	Up	[46]
HMGCR	3-Hydroxy-3-methylglutaryl-coenzyme A reductase	Cholesterol biosynthesis	HFD	6	Male Golden Syrian hamsters	Up	[43]
HMGCS1	3-Hydroxy-3-methylglutaryl-coenzyme A synthase 1	Cholesterol biosynthesis	HFD	16	Male Wistar rats	Down	[44]
IRS-2	Insulin receptor substrate-2	Controls various cellular processes by insulin	HFD	12	Male Wister rats	Down	[47]
LDLR	Low-density lipoprotein receptor	Clearance of cholesterol	HFD	9	Male SD rats	Down	[42]
				6	Male Golden Syrian Hamsters	Down	[43]
LEP	Leptin	Regulation of energy balance and body weight control	HFD	8	Male Wistar rats	Up	[46]
MTMR4	Myotubularin-related protein 4	Phosphatase activity and protein serine/threonine phosphatase activity	HFD	16	Male Wistar rats	Up	[44]
PPAR-A	Peroxisome proliferator-activated receptor alpha	β-Oxidation and energy expenditure	HFD	9	Male SD rats	Down	[42]
				16	Male Wistar rats	Down	[44]
				24	Male C57BL/6J mice	Down	[45]
PPAR-γ	Peroxisome proliferator-activated receptor gamma	Controls the peroxisomal β-oxidation pathway of fatty acids	MCD	3	C57BL/6J mic	Up	[48]
SREBF1	Sterol regulatory element-binding protein 1 (isoform SREBP-1a)	Stimulates both lipogenic and cholesterogenic gene expression	HFD	16	Male Wistar rats	Up	[44]
				9	Male SD rats	Up	[42]
				24	Male C57BL/6J mice	Up	[45]
			MCD	4	db/db mice	Down	[37]
					db/m mice	Down	
	Sterol regulatory element-binding protein 1 (Isoform SREBP-1c)	Primarily controls the expression of the lipogenic gene			db/db mice	Up	
					db/m mice	Down	
SCD-1	Stearoyl-CoA desaturase-1	Plays an important role in lipid biosynthesis	MCD	4	db/db mice	Down	
					db/m mice	Down	
			HFD	16	Male Wistar rats	Up	[44]

**Table 3:** Gene expression profiles of hepatic inflammation in HFD and MCD diet-fed animal models.

Gene Symbol	Nomenclature	Role	Diet Type	Duration (Weeks)	Animal Model	Gene Expression	Reference
CFH	Complement component factor H	Plays an essential role in maintaining a well-balanced immune response	HFD	16	Male Wistar rats	Up	[44]
COX-2	Prostaglandin-endoperoxide synthase 2 (cyclooxygenase-2)	A particular role in the inflammatory response	HFD	8	Female SD rats	Up	[30]
CXCL1	Chemokine (C-X-C motif) ligand 1	Plays a role in inflammation and as a chemoattractant for neutrophils	HFD	16	Male Wistar rats	Up	[44]
CXCL14	Chemokine (C-X-C motif) ligand 14	Chemotactic for B-lymphocytes	HFD	16	Male Wistar rats	Down	
IL-1β	Interleukin-1 beta	Induces prostaglandin synthesis, neutrophil influx and activation, and T cell activation	MCD	2	Male SD rats	Up	[51]
				5	Male C57BL/6N mice	Up	[49]
IL-6	Interleukin-6	Regulation of the immune response	MCD	2	Male SD rats	Up	[51]
				5	Male C57BL/6N mice	Up	[49]
MCP-1	Monocyte chemoattractant protein-1	Exhibits chemotactic activity for monocytes and basophils	MCD	5	Male C57BL/6N mice	Up	
NF-κB1	Nuclear factor-kappa B subunit 1	Stimulates many biological processes such as inflammation, immunity, differentiation, cell growth, tumorigenesis, and apoptosis	MCD	5	Male C57BL/6N mice	Up	
iNOS2	Inducible nitric oxide synthase	Involved in inflammation, enhances the synthesis of proinflammatory mediators, such as IL-6 and IL-8	HFD	8	Female SD rats	Up	[30]
TGF-β1	Transforming growth factor-beta 1	Multifunctional protein that regulates the growth and differentiation of various cell types	MCD	2	Male SD rats	Up	[51]
				5	Male C57BL/6N mice	Up	[49]
				12	Male SD rats	Up	[52]
TNF-α	Tumor necrosis factor-alpha	A key mediator of cell death, and induces insulin resistance	HFD	6	Male SD rats	Up	[50]
				24	Male C57BL/6J mice	Up	[45]
				8	Female SD rats	Up	[30]
				16	Male C57BL/6 mice	Up	[53]
				25	Male C57BL/6 mice	Up	[22]
			MCD	2	Male and female C57BL/6J mice	Up	[54]
				4	C57BL/6 mice	Up	[55]
				2	Male SD rats	Up	[51]
				5	Male C57BL/6N mice	Up	[49]

**Table 4:** Gene expression profiles of hepatic oxidative stress in HFD and MCD diet-fed animal models.

Gene Symbol	Nomenclature	Role	Diet Type	Duration (Weeks)	Animal Model	Gene Expression	Reference
AOX	Alternative oxidase mitochondrial precursor	Catalyzes the cyanide-resistant oxidation of ubiquinol	MCD	4	db/db mice	Down	[37]
					db/m Mice	Up	
CAT	Catalase	Protects cells from the toxic effects of hydrogen peroxide	HFD	8	Female SD rats	Down	[30]
CHOP (DDIT3)	C/EBP homologous protein (DNA damage-inducible transcript 3)	Endoplasmic reticulum (ER) stress response	HFD	25	Male C57BL/6 mice	Up	[22]
CPT-1	Carnitine O-palmitoyltransferase 1	Plays role in mitochondrial uptake of long-chain fatty acids	MCD	4	db/db mice	Up	[37]
				4	db/m mice	Up	
CPT-2	Carnitine O-palmitoyl transferase 2, mitochondrial	Intra-mitochondrial synthesis of acylcarnitines	MCD	4	db/db mice	Up	
				4	db/m mice	Up	
GAB1	GRB2-associated binding protein 1	Plays a role in FGFR1 signaling	HFD	16	Male Wistar rats	Up	[44]
GADD45G	Growth arrest and DNA-damage-inducible, gamma	Mediates activation of stress-responsive MTK1/MEKK4 MAPKKK	HFD	16	Male Wistar rats	Up	
Gp91phox (CYBB)	Cytochrome B-245, beta polypeptide	A critical component of the membrane-bound oxidase of phagocytes that generates superoxide	HFD	24	Male C57BL/6J mice	Up	[45]
GPX1	Glutathione peroxidase	Protects from oxidative breakdown	HFD	8	Female SD rats	Down	[30]
LCAD	Long-chain specific acyl-CoA dehydrogenase, mitochondrial	Catalyzes the first step of mitochondrial fatty acid beta-oxidation	MCD	4	db/db mice	No change	[37]
				4	db/m mice	Up	
L-FABP	Fatty acid-binding protein, liver	Plays a role in lipoprotein-mediated cholesterol uptake	MCD	4	db/db mice	Down	
				4	db/m mice	Down	
NFE2L2	Nuclear factor, erythroid 2-Like 2	Transcription factor	HFD	16	Male Wistar rats	Up	[44]
P22phox (CYPA)	Cytochrome B-245, alpha polypeptide	Critical component of the membrane-bound oxidase	HFD	24	Male C57BL/6J mice	Up	[45]
P47phox (NCF1)	Neutrophil cytosolic factor 1	Activation of the latent NADPH oxidase (necessary for superoxide production)	HFD	24	Male C57BL/6J mice	Up	
PERK	Protein kinase R (PKR)-like endoplasmic reticulum kinase	Plays a role in the early steps of protein synthesis	HFD	25	Male C57BL/6 mice	Down	[22]
SIRT1	Sirtuin 1 (silent mating type information regulation 2 homolog)	Deacetylase, ADP-ribosyl transferase, and other deacylase activities	HFD	12	Male Wistar rats	Down	[56]

**Table 5:** Gene expression profiles of hepatic fibrogenesis in HFD and MCD diet-fed animal models.

Gene Symbol	Nomenclature	Role	Diet Type	Duration (Weeks)	Animal Model	Gene Expression	Reference
ACTA2 (ASMA)	Anti-smooth muscle actin	Activation to myofibroblast-like cell	HFD	25	C57BL/6 mice	Up	[22]
COL1A1	Collagen type 1 alpha 1	Fibrillar forming collagen	HFD	25	C57BL/6 mice	Up	[22]
				24	Male C57BL/6J mice	Up	[45]
			MCD	2	Male SD rats	Up	[51]
				17	Male SD rats	Up	[52]
COL1A2	Collagen type I alpha 2		HFD	24	Male C57BL/6J mice	Up	[45]
COL4A1	Collagen type IV alpha 1		HFD	24	Male C57BL/6J mice	Up	[45]
HGF	Hepatocyte growth factor	Hepatotropic factor, which acts as a growth factor	HFD	25	C57BL/6 mice	Up	[22]
LUM	Lumican	Extracellular matrix structural constituent	HFD	25	C57BL/6 mice	Up	[22]
MMP-13	Matrix metalloproteinase-13	Degradation of extracellular matrix proteins	MCD	2	Male SD rats	Up	[51]
MMP-9	Matrix metalloproteinase-9		MCD	2	Male SD rats	Up	[51]
PAI-1	Plasminogen activator inhibitor 1	Inhibitor of tissue-type plasminogen activator (PLAT) and urokinase-type plasminogen activator (PLAU)	HFD	24	Male C57BL/6J mice	Up	[45]
SOCS1	Suppressor of cytokine signaling 1	Prevents uncontrolled cytokine signaling	MCD	2	Male SD rats	Up	[51]
TIMP-1	Tissue inhibitor matrix metalloproteinase 1 (TIMP Metallopeptidase Inhibitor 1)	Inhibitor of collagenases by forming one to one complexes	MCD	17	Male SD rats	Up	[52]
			HFD	25	C57BL/6 mice	Up	[22]
TGF-β1	Transforming growth factor-beta 1	Acts as a regulator of extracellular matrix storage	HFD	25	C57BL/6 mice	Up	[22]
				24	Male C57BL/6J mice	Up	[45]

## Data Availability

Not applicable.
